# Pension and state funds dominating biomedical R&D investment: fiduciary duty and public health

**DOI:** 10.1186/s12992-019-0490-x

**Published:** 2019-11-06

**Authors:** Slavek Roller

**Affiliations:** Goethe University Frankfurt, Theodor-W.-Adorno-Platz 1, 60323 Frankfurt, Germany

**Keywords:** Pharmaceuticals, Drug development, Access to medicines, R&D investment, Corporate governance

## Abstract

**Background:**

Who benefits from the commercial biomedical research and development (R&D)? Patients-consumers and investors-shareholders have traditionally been viewed as two distinct groups with conflicting interests: shareholders seek maximum profits, patients - maximum clinical benefit. However, what happens when patients are the shareholders? With billions of dollars of public risk capital channeled into the drug development industry, analysing the complex financial architecture and the market for corporate control is essential for understanding industry’s characteristics, such as pricing strategies or R&D priorities.

**Results:**

Adding investments by governmentally-mandated retirement schemes, central and promotional banks, and sovereign wealth funds to tax-derived governmental financing shows that the majority of biomedical R&D funding is public in origin. Despite this, even in the high-income countries patients can be denied access to effective treatments due to their high cost. Since these costs are set by the drug development firms that are owned in substantial part by the retirement accounts of said patients, the complex financial architecture of biomedical R&D may be inconsistent with the objectives of the ultimate beneficiaries.

**Conclusions:**

The divergence in economic and public health performance of the drug development industry is resultant from its financial underwriting by enormously expanded pension schemes, governmentally mandated to represent the interests of “captive” beneficiaries, as well as similar policymaker-designed funding flows, whose standards of transparency, accountability and representation are substantially lower than that of governments themselves. Strengthening those elements of institutional design and thus ensuring active responsible shareholding in the interest of the patients-savers is an under-utilised, but potentially high-impact opportunity for advancing public health.

**Electronic supplementary material:**

The online version of this article (10.1186/s12992-019-0490-x) contains supplementary material, which is available to authorized users.

## Background

Who benefits from the commercial biomedical research and development (R&D)? On a population level there are two groups of agents standing to benefit from the biomedical R&D: the patients/public who accrue the social value of new medical technologies, and the producers/owners who accrue the economic value from the sales of such technologies. Public health literature has traditionally treated the two as distinct groups with opposing interests. In contrast, in this study I demonstrate with the growth of public and quasi-public investors, such as national and occupational retirement schemes, the majority of biomedical R&D funding is public in origin. Furthermore, how the biomedical R&D’s value is captured by the public in their role as savers and how it is captured by the public in their role as patients is policymaker-designed, institutionalised and regulated.

After Italy became the last of high-income jurisdictions to abandon express prohibition against patenting of drugs in 1978 ([1], *316*), the institutional distribution system has ensured that each of the roles, patients-consumers and investors-shareholders, capture a positive value from the biomedical innovation. By 2010s this arrangement is no longer universal. The products of biomedical R&D are getting priced out of access even in high-income countries, the countries, whose savers had provided the input capital for that R&D - in a significant part via institutional investing channels, such as retirement schemes. Institutional investors have a fiduciary duty to act in the best interests of their savers-beneficiaries. How can then a situation be explained in which savers’ pension assets are invested into companies whose medicines are not available to said savers due to the high cots?

## Methods

To detail the complex financial architecture of modern drug development, I use a three-step approach. Firstly, I use macrofinancial statistics to demonstrate how savings of citizens in high-income countries are institutionally channeled into the stock market, including its healthcare sector via retirement schemes as well as other public funds. I show that this provides at least one third of input capital in the drug development industry. Secondly, I survey the outputs of the drug development industry (medicines), which are priced out of markets, from which the input capital is sourced. Thirdly, I verify the hypothesis inferred from the macrofinancial statistics that public and quasi-public institutional investors, such as national and occupational retirement schemes, are major investors in commercial drug developers. To this end I trace the shareholdings in the companies producing the medicines reviewed in step two within the disclosures of portfolio holdings of individual public and quasi-public institutional investors in the jurisdictions that limit access to said medicines.

This methodology is intended to draw a full circle in the process of modern drug development: from the provision of input capital, where a significant share is allocated by institutional investors (predominantly pension funds), to the output products’ admission into the market in the jurisdiction of said investors. By the end of this process, the values of the biomedical R&D are expected to be realised - both economic value, and the social value - by those who provided the original input capital. Yet the case studies demonstrate situations where the social value is not realised as intended: the companies set prices so high that the national health insurers deny reimbursement. Such cases are more exceptions than they are the rule, but precisely by studying the outliers can we better understand the system and the range of possible outcomes that it is able to produce.

### Accounting for public and quasi-public funding of biomedical R&D beyond direct government allocations

It has been estimated that of the total $265 billion spent annually on biomedical research worldwide, over a third - $103 billion comes from public sources [2]. Nevertheless, as public input capital is allocated predominantly into early stage research, nearly all output - medicines - is ultimately brought to the market by private firms. Importantly, these firms are not independent agents. They have owners-shareholders to report to. Until the end of the previous century the major type of owners-shareholders were individual households. At the turn of the millennium, however, they have been displaced by institutional investors, the largest of which are public retirements schemes or quasi-public funds, such as occupational pensions. In this section I make use of macrofinancial data to document the large and growing public ownership in private^1^ firms.

Estimates for the aggregate household wealth locked in mandatory or tax-incentivised pension schemes are taken from the Organisation for Economic Co-operation and Development (OECD) “Pension Markets in Focus” 2017 datasets [44]. The total investment of pension providers in OECD countries have grown from $25.3 trillion in 2006 to $38.1 trillion^2^ in 2016. The proportion of this wealth allocated to shares of exchange-listed companies can also be calculated using the OECD data. As shown in Table 1, $14.3 trillion was allocated via outright investment into shares as well as via investment into shares through collective investment schemes (mutual funds). Some countries only have total assets reported without additional data on asset allocation. Where the data on asset allocation to shares was not available, a weighted average of 27% for the available countries was used. This same approach was used for calculating public equities ownership within collective investment schemes, whereby a weighted average of 33% was used to fill the gaps.

**Table 1:** Accounting for Share Ownership by OECD Retirement Schemes [44]

Country	Total Investment by Retirement Schemes, $trillion	Allocated to Equities, %	Allocated to Mutual Funds, %	Allocated to Equities within Mutual Funds, %	Total Allocation toEquities, $trillion
United States	25.127	31	33	–	10.581
Australia	1.523	51	0	–	0.778
Canada	2.404	23	38	16	0.695
Netherlands	1.335	14	53	48	0.524
United Kingdom	2.274	14	27	–	0.517
Switzerland	0.904	9	56	38	0.275
Denmark	0.612	22	8	–	0.150
Sweden	0.389	15	62	–	0.140
Japan	1.355	9	0	–	0.128
France	0.230	–	–	–	0.086
Chile	0.174	8	38	68	0.059
Finland	0.135	37	0	–	0.050
Ireland	0.118	33	0	–	0.039
Poland	0.041	83	0	–	0.034
Italy	0.165	13	11	52	0.032
Mexico	0.157	10	14	72	0.032
Spain	0.164	11	18	–	0.028
Korea	0.365	3	5	–	0.018
Israel	0.177	8	4	63	0.018
New Zealand	0.045	20	32	–	0.014
Norway	0.037	15	36	57	0.013
Belgium	0.031	9	72	47	0.013
Germany	0.224	0	45	11	0.011
Iceland	0.032	16	18	85	0.010
Austria	0.022	33	0	–	0.007
Turkey	0.035	12	0	–	0.004
Portugal	0.021	7	28	42	0.004
Estonia	0.004	3	55	56	0.001
Hungary	0.005	8	26	–	0.001
Slovak Republic	0.010	2	19	–	0.001
Latvia	0.003	1	38	50	0.001
Luxembourg	0.002	0	50	50	0.000
Slovenia	0.003	1	21	–	0.000
Greece	0.001	7	25	–	0.000
Czech Republic	0.016	0	2	–	0.000
TOTAL	38.140				14.265

Source: Author’s calculations based on OECD Pension Markets in Focus 2017 dataset

According to the Word Bank, the total market capitalisation of the OECD countries was $45.9 trillion in 2016 [53]. Assuming that OECD pension schemes allocation into non-OECD stocks is compensated by capital allocations into OECD stocks by non-OECD pension schemes, such as those of Brazil ($0.4 trillion in total investment by pension schemes) and Singapore ($0.2 trillion), $14.3 trillion or 31% share of the OECD stock market is controlled by the OECD pension schemes. This share for all OECD members is slightly lower than the 37% ownership share of the US stock market by the US retirement accounts and plans [54]. In the US, Rosenthal & Austin used the Federal Reserve’s “Financial Accounts of the United States” data to document how retirement schemes displaced households to become the main owners of US stocks at the turn of the millennium.

The 31% estimation based on the OECD data is likely an underestimate. While OECD has recently expanded their calculation methodology from covering only pension funds to covering pension funds and retirement schemes managed by insurance companies, such data coverage is incomplete. Alternative survey estimates by the International Monetary Fund (IMF) [55] from 2010 put the origin of assets managed by institutional investors at 33% retail (that is, households), 26% pension funds, 18% insurance companies (how much of this is in retirement products was unspecified), ~ 2.5% each for endowments and banks, 1.5% sovereigns with the rest unspecified. Thus, OECD, IMF and US Federal Reserve-based calculations broadly agree that a third of the stock market is controlled by retirement accounts of various types.

Full consolidation accounting of public and quasi-public investment in biomedical R&D beyond retirement schemes requires further incorporation of governmental shareholdings, central bank purchases of corporate securities, sovereign wealth fund investments and capital injections by promotional or development banks. Capital allocations by sovereign wealth funds grew from $0.6 trillion in 2002 to $5.6 trillion in 2016. Of these, $2.1 trillion are in public equities [5] while $0.5 trillion are allocated to corporate stocks and bonds by the central banks in the Euro area, Japan and Switzerland.^3^ Public shareholding extends beyond countries that control large sovereign wealth funds (Qatar, Norway) to those that do not (UK, France), but have otherwise accumulated substantial ownership of private firms, for instance via recapitalisation of banks during the global financial crisis. Bank recapitalisations extended the investment reach of such countries through the ownership of the acquired banks in non-financial firms. Peetz & Murray [56] in a study of 250 largest industrial corporations and 50 largest financial corporations traced 34% of shareholding to private financial firms and 17% to the governments of the UK, China, Qatar, Japan, France, Norway, USA, Belgium and Germany. Finally, state-owned promotional^4^ banks, such as the European Investment Bank or the Business Development Bank of Canada also allocate capital into commercial biomedical R&D, albeit in smaller volumes of billions USD.

### Connecting the (lacking) output benefits to those directly providing the input capital

According to the data compiled by pharmaceutical associations in Canada and Australia, over 90% of new medicines (new active ingredients) registered after 2011 are reimbursed by health insurance in Japan, while in Australia, Canada and Portugal this share is below 50% [6, 7]. In Australia this results in 100 new active ingredients being approved for marketing, but not reimbursed. New Zealand has the lowest reimbursement rate: only 22% of new active ingredients are covered by the national health insurance. The OECD average is 61%. In some cases where reimbursement status was granted, it occured after - considerable delay. In Canada for instance, five new oncology drugs approved between 2003 and 2011 for the treatment of advanced solid tumours were accepted for reimbursement between 1.5 and 5 years after the regulatory approval, resulting in an estimated loss of 1.7 thousands life-years among the patients affected [8]. Furthermore, a drug may also be reimbursed only for some of the approved indications, but not for others [9].

For this study I select cases of the universal denial of coverage within a given jurisdiction for defined patient populations based on cost considerations. These cases apply to two major types of medicines that have been the focus of the drug development industry in the past decade: cancer drugs and orphan drugs (drugs for rare diseases). Together these two categories make up the majority of new active ingredients coming to the market. For instance, out of 42 new active ingredients approved by the European Medicines Agency in 2018, 11 (26%) were for cancer, and 12 (29%) for non-cancer rare diseases [10].

Consider bevacizumab, one of the recent solid tumor medicines developed by Roche. Despite appeals from patient advocacy groups, the Canadian Agency for Drugs and Technologies in Health (CADTH) concluded that the drug was not cost-effective at the submitted price for the treatment of cervical cancer, metastatic colorectal cancer, and ovarian cancer. The CADTH cited among other reasons, high cost and unknown treatment duration (see Additional file 1). As healthcare policy in Canada falls under provincial purview, access to bevacizumab currently depends on province the patient lives in: British Columbia, Saskatchewan and Manitoba have public access while other provinces don’t [11].

Likewise, while accepting the evidence for statistically significant improvements after treatment with bevacizumab in metastatic colorectal cancer patients, the UK’s National Institute for Health and Care Excellence (NICE) advised against reimbursement. It also denied reimbursement in any other oncology use for which the drug had been approved. Similarly, New Zealand’s National Pharmaceutical Management Agency did not reimburse bevacizumab, except for use in ophthalmology [12].

The situation is not unique for cancer medicines, access limitations also apply to breakthrough drugs for rare diseases, such as asfotase alfa and eculizumab by Alexion Pharmaceuticals.

Eculizumab, an effective treatment for rare blood disorders is priced at about $0.5 million per patient per year [13]. It was denied reimbursement in Canada, New Zealand (“The main reason for this decision was that the price being sought by Alexion Pharmaceuticals (the supplier) is too high for PHARMAC [the Pharmaceutical Management Agency] to justify funding”), and the Netherlands, where the Zorginstituut Nederland (National Health Care Institute) estimated the life-long eculizumab treatment to be about 15 million Euro [14]. Zorginstituut Nederland subsequently authorised a 3-month course of eculizumab for reimbursement within the nationwide CUREiHUS study [15].

Occasionally, a reimbursement denial indicates the extent, to which the decision is driven by cost. CADTH Final Recommendation report on the Alexion Pharmaceuticals’ another breakthrough medicine, asfotase alfa, determined that “the annual cost will exceed $1 million for patients weighing more than 20 kg”, and as a result, “even with a price reduction of 90%, asfotase alfa is unlikely to be a cost-effective treatment option for HPP [hypophosphatasia]”. More frequently however, the reimbursement denial decisions only include less specific formulations, citing the price “among other reasons” or as “the main reason”.

Contrast this approach to France, a jurisdiction, where cost-effectiveness is not considered for post-marketing appraisal of medicines [16]. The French equivalent of CADTH and NICE, the National Authority for Health (HAS), determined that bevacizumab offers improvement over available therapies in colorectal and ovarian cancer, advanced and/or metastatic renal cell carcinoma; persistent, recurrent or metastatic cervical cancer, as well as in combination with paclitaxel for the treatment of breast cancer. Thus, HAS recommended bevacizumab and eculizumab for inclusion on the list of reimbursable products for hospital use.

In the largest market for pharmaceutical products, the United States, no national health technology assessment body exists. The Office of Technology Assessment was defunded by Congress in 1995 [17] and the coverage of bevacizumab and eculizumab varies with the health insurance provider. Regulatory decisions limiting access to bevacizumab, eculizumab and asfotase alfa across OECD are provided in the Additional file.

### Tracing savers’ retirement schemes investments in companies whose medicines are not reimbursable in the jurisdictions where said savers reside

Nowhere is the public nature of the major institutional investors is evidenced better as in their disclosure of portfolio holdings. Financial institutions are typically known for their secrecy. A bank or a private insurance firm would never disclose in which securities its clients’ portfolios are invested. But where the institutional investor’s mandate is to serve the public or a large segment of the public (such as all members of a specific profession, as in occupational retirement schemes), then it discloses the destination of its investments - publicly.

While disclosure details vary, by collecting the data from the funds with the highest transparency standards - those disclosing ownership stakes separately for every company they invest in - it is possible to reconstruct that Roche (developer of bevacizumab) and Alexion Pharmaceuticals (developer of eculizumab and asfotase alfa) are owned in part by the *Canada Pension Plan Investment Board (CPPIB)*, *Canada’s British Columbia Investment Management Corporation (*provider of investment services to British Columbia’s public sector), *The New Zealand Superannuation Fund (NZ Super Fund)*, Dutch *ABP* - pension fund for employees in the government and education sectors, UK’s *Strathclyde Pension Fund* (only Alexion), UK’s *West Yorkshire Pension Fund* (only Roche). Holdings data for the reviewed funds is presented in Table 2.^5^

**Table 2:** Holdings of public and quasi-public institutional investors in Roche and Alexion Pharmaceuticals - companies whose drugs were denied reimbursement by health insurers in high-income countries due to high prices

Fund	Assets under management, USD million	Holdings in Hoffmann-La Roche Ltd. (marketer of bevacizumab), USD million	Holdings in Alexion Pharmaceuticals Inc. (marketer of eculizumab), USD million	Date
ABP (Netherlands; pension fund for employees in the government and education sectors)	498250	649	134	3/2018
Canadian Pension Plan Investment Board	238160	689	41	3/2017
British Columbia Investment Management Corporation (Canada)	101890	109	25	3/2017
New Zealand Superannuation Fund	27670	43	6	6/2017
Strathclyde Pension Fund (UK)	28230	–	6	12/2017
West Yorkshire Pension Fund (UK)	19500	43	–	3/2017
*Swiss National Bank (Switzerland)*	*92585 (US equities only)*	*–*	*98*	*2/2018*
*European Central Bank (EU)*	*185140*	*9260 in health & life sciences corporate bonds in total, mostly Bayer, Merck (Germany), Novartis, Roche and Sanofi. Holdings per firm not disclosed.*	*–*	*4/2018*

*Sources:* [24–32]. Holdings of the pension funds are shown in roman font; holdings of the central banks - in italic font.

Many more retirement schemes in Canada, New Zealand, UK, the Netherlands and other OECD countries are also shareholders in Roche and Alexion, though not all of their holdings can be verified since not all retirement schemes are as transparent as those listed above. For instance, another major Canadian pension fund, *Caisse de dépôt et placement du Québec,* does not disclose firm-level holdings at all, only stating that CAD 40 billion is invested in global equities [23]. The gaps in retirement schemes’ holdings disclosure currently precludes a more detailed systematic analysis.

## Results

About a third of the stock market is controlled by retirement accounts of various types. Most of the healthcare firms stock purchases by retirement accounts are non-productive investments into existing intellectual property rents [4] rather than productive investment into new R&D. However, if we assign the credit for new R&D investment according to the stock ownership share, a third of the $162 billion annually spent by the industry worldwide can be attributed to institutional investors legally mandated to represent “captive” beneficiaries. Such institutional investors include public and quasi-public pension funds (for instance, national and occupational retirement schemes), sovereign wealth funds, banks with large governmental shareholding, central banks, as well as state-owned development banks.

One hundred sixty-two billion dollars spent by the industry on biomedical R&D is gross risk capital. Indirect support funding mechanisms for commercial biomedical R&D, such as tax credits and matching funds, are estimated to range from 17% of the total R&D funding, according to industry-sponsored studies [59], to up to 59% in Canada, where $0.4 billion net R&D investment by industry in 2007 was amplified by $0.6 billion tax subsidy by 2010. [60].

**Fig. 1:**
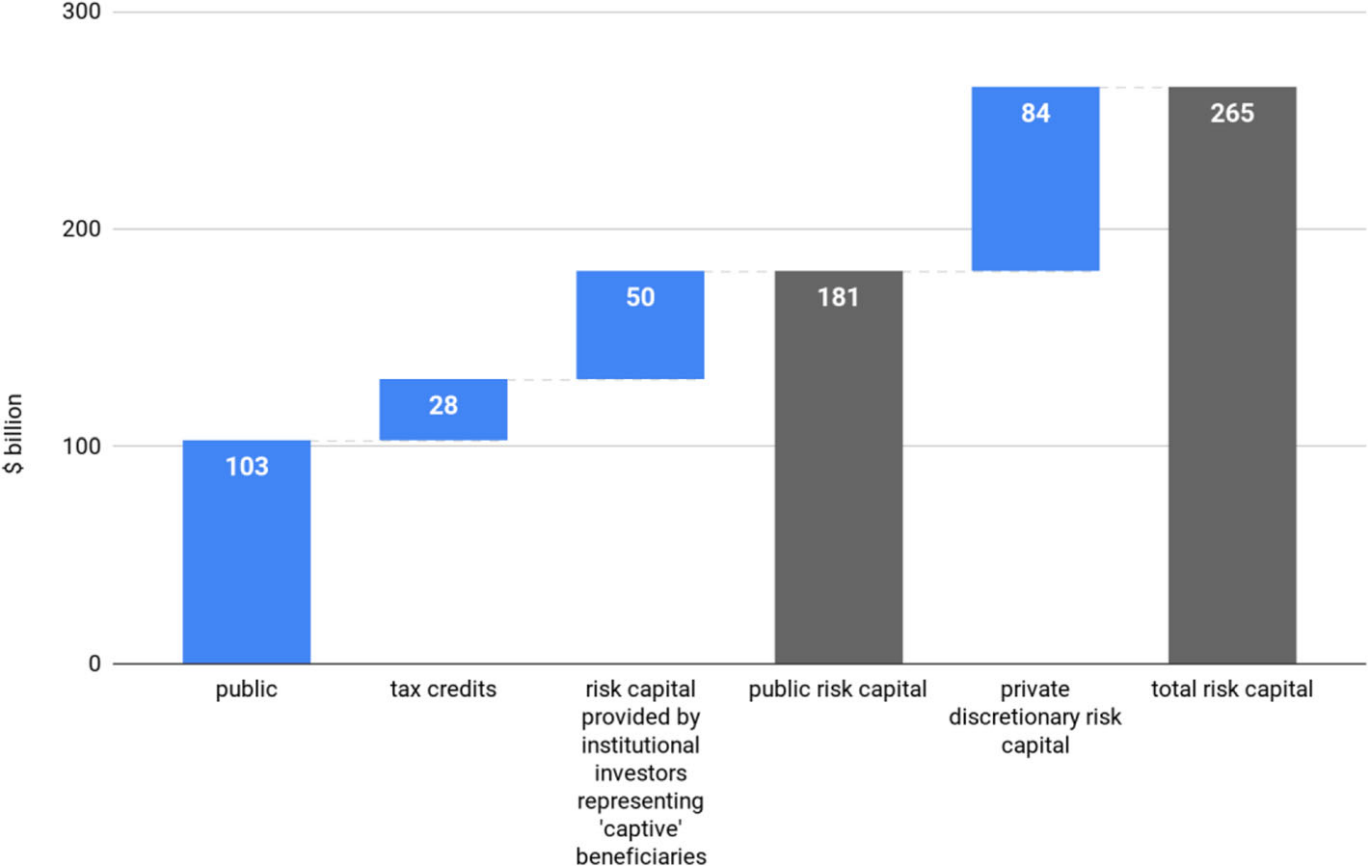
Risk capital for biomedical R&D by source. *Sources: Author’s calculations. The split between public and industry funding is based on Moses et al.* [2]*. Tax credit estimate of 17% is based on DiMasi et al.* [61]*. Risk capital provided by institutional investors includes investments by pension funds* [46]*, sovereign wealth funds* [6] *and central banks (*[59]*;* [31]*;* [32]*).*

To calculate net risk capital I apply a conservative 17% tax deduction on the gross $162 billion of annual R&D spending by industry. The net $134 billion is then attributed to institutional and discretionary investors, proportionately to their ownership in drug development firms. Thus 31% is attributed to pension funds, 5% to sovereign wealth funds and 1% to major OECD central banks. Together these institutional investors representing “captive” beneficiaries ultimately fund $50 billion in commercial biomedical R&D annually. Using this methodology, more than two thirds of the risk capital for biomedical R&D can be traced to the public source (see Fig. 1).

Commercial decisions, such as setting prices on new products are outsourced by principals providing risk capital to agents-executives who deploy this capital in the principals’ interest. As the case studies presented in this study demonstrate, executives can price new products above the cost-effectiveness thresholds instituted by national governments in an attempt to maximise overall profit by selling highly-priced products in other jurisdictions. As a result, access to medicine depends on the citizenship of the patients. This is not new in itself: during a well-documented HIV/AIDS crisis, citizens of low-income countries in Africa and Asia could not access effective medicines available in high-income countries [18]. What is new is that even patients in the high-income countries are denied access to effective treatments due to their high cost, all the while these costs are being set by the drug development firms, owned in substantial part by the retirement accounts of said patients, and benefitting from direct and indirect governmental support.

## Discussion

### Financial architecture of drug development

The results demonstrate significant provision of risk capital for biomedical R&D by public schemes beyond direct allocations by governments. Specifically, savers are either legally required or strongly incentivised to contribute a share of their earnings into retirement funds (see Clark & Monk [3] for the discussion of "captive" beneficiaries). The administrators of these retirement funds appoint investment managers to invest these savings. Investment managers, who sometimes are internal professionals at the retirement schemes, but more often external service provider companies, contract investment index publishers to provide a benchmark index, around which an investment portfolio will be built and against which the portfolio returns are going to be evaluated. And finally once the savings have been invested into individual firms, appointed executives of those firms allocate capital into R&D projects, which ultimately result in new products - medicines.

Between the retirement schemes administrators (or other managers of large public funds, such as portfolio managers at the central banks and sovereign wealth funds), investment management firms, index providers, and appointed executives at the portfolio firms there are contractual agreements and powerful incentive systems. All the agents except for the closest one to the principal (pension board members are closest to savers-principals, see Table 3) have compensation structures that include a proportion of assets under management, or a multiplier based on financial performance, or both. As a result, their compensation scales superlinearly with the underlying corporate earnings, that is, it increases consistently at a nonlinear rate greater than one for one. Consider the case of Canadian savers and Alexion Pharmaceuticals. Annual board member retainer fee at the Canadian Pension Plan Investment Board is $50 thousand. The total compensation of CPPIB’s chief executive in 2018 - $4 million (= base salary × incentive target × performance multiplier) [19]; chief executive of the investment manager BlackRock - $24 million [20]; chief executive of Alexion Pharmaceuticals - $ 16 million [21].

**Table 3:** Savers-principals and their agents

Distance from principals	Agents	Financial flows order of magnitude^a^	Authority by	Incentive	Compensation	Geography
Principals	Savers	Thousands	Asset ownership	Welfare	May be based on financial performance^b^. Linear scaling	Dispersed
1	Pension fund board	Billions	Election or political appointment	Political	Fixed retainer fee	Concentrated: regional or national
2	Pension fund executives		Appointment by pension fund board	Financial return	Based on financial performance. Superlinear scaling	
3	Investment managers	Trillions	Appointment by pension fund executives	Financial return	Based on financial performance. Superlinear scaling	Concentrated: global financial centres
4	Corporate directors	Millions	Appointment by investment managers’ vote on shares	Financial return	Based on financial performance. Superlinear scaling	Concentrated: global economic hubs

^a^*Annual pension contributions in high-income countries amount to several thousands to tens of thousands of USD. Pension funds can range from several billion to several hundred billion USD, while the largest investment managers have several trillion USD under management. They invest in stock-exchange-listed corporations and vote their shares to elect corporate executives. Corporate executives then have authority over smaller operational budgets that can amount to hundreds of million USD (for instance, Alexion Pharmaceuticals annual R&D budget in 2018 was $730 million* [22]).

^b^ *Savers can benefit from their pension fund’s strong financial performance in other ways, for instance, through a reduction of monthly contributions*

Alexion’s “Letter from the leadership and compensation committee chair” included into the Proxy Statement, which solicits shareholder votes for the 2019 Annual Meeting states that “The Board of Directors, the leadership team and our employees are all committed to deliver value to our patients, and through this, deliver long-term value for our shareholders.” In practice, “value delivered to patients” is not part of a contractual incentive system: 65% of Annual Cash Incentive Compensation for executives at Alexion is based on financial metrics including Revenue, Operating Margin, Earnings Per Share, and Free Cash Flow, further 35% of our goals based on strategic objectives (strong commercial performance, improved operating margins, receipt of marketing approval) [21]. No “value delivered to patients” metrics, such as number of patients treated, clinical value added, or quality-adjusted life-years gained are included. Similarly, further up in the financing chain only financial metrics are used by investment managers to formally assess drug development companies, by asset owners executives (such as pension fund investment staff) to formally assess their investment managers, and by the asset owners bards to formally assess their own executives.

With four levels of intermediaries involved, the preferences of the ultimate capital providers may be subverted by the preferences of the intermediaries themselves. This can lead to financial underwriting of business practices that may not represent the interests of savers providing the capital. Healthcare sector investing is a case in point. Trillions of dollars in pension assets are domiciles in high-income countries whose citizens have democratically voted on healthcare systems based on a solidarity principle, whereby a person’s health insurance premium does not depend on their state of health. The goal of such healthcare systems is to ensure universal coverage. In contrast to the provision of healthcare, the provision of healthcare technology is market-based with no embedded solidarity principle. Accordingly, a drug company may find it optimal to set such a price to its products that would exclude some patients from access, while maximising the total revenue from other patients (in other jurisdictions). This can lead to a contradictory situation where the same person is financing - via her tax contributions - a government seeking to ensure universal healthcare provision,^6^ and at the same time is financing - via her pension contributions - corporations expressly employing limited access pricing strategies as a way of maximising profits.

### Implications for fiduciary duty

As governments in high-income countries incentivise or mandate retirement savings, the cost considerations of administering these savings result in pooling and centralisation. Assets under management of individual funds reviewed in Table 2 range from $20 billion to $500 billion (to put this in perspective, recall that the entire global volume of biomedical R&D investment annually is $265 billion, approximately equal to the CPPIB assets under management). Investment managers - private firms whom asset owners, such as CPPIB, contract to manage their assets - are more concentrated still. The largest such providers of investment management services are BlackRock, a firm that manages $5+ trillion (of which $2.6 trillion is in shares of stock-exchange-listed companies), and Vanguard, managing $4 trillion (of which $2.2 trillion is in shares^7^). BlackRock is employed, among others, by CPPIB, NZ Super Fund and West Yorkshire Pension Fund (UK). An example of Vanguard’s institutional client is the California State Teachers’ Retirement System (CalSTRS) [42].

BlackRock and Vanguard, together with the third largest investment manager, State Street, (the “Big Three”) collectively represent an average of 25% of the shares voted in director elections at 500 largest US-based companies that constitute S&P 500 stock market index [34, 35]. S&P 500 includes 8 biotechnology (Alexion Pharmaceuticals among them) and 9 pharmaceutical companies producing human medicines. The Big Three thereby have more power over the state and the future of healthcare and biomedical science than the National Institutes of Health and the European Commission. While the latter two are under untiring scrutiny of the medical and scientific communities, the former two remain in the shadows. The Big Three and Vanguard are “passive” investors that closely follow market-capitalisation-based stock market indices and use private “engagements” as a preferred tool of exercising corporate control. For instance, during the year ending in mid-2015, BlackRock and Vanguard performed over 1500 and 800 private “engagements” with companies held in its portfolio respectively [43]. It is not possible to audit such private engagements.

Responsible stewardship practiced by investment management firms, which includes engagements and other forms of shareholder activism, is driven by the fiduciary duty of institutional asset owners that allocate capital to investment management firms. Fiduciary duty - a responsibility of the asset managers to act in the interests of the beneficiaries - has no legal definition [36, 37]. Traditionally, corporate directors and asset managers have both perceived themselves to have a fiduciary duty to maximise shareholder value [38]. More recently, communications by some of the largest retirement scheme managers suggest broadening of the interpretation of what fiduciary duty actually entails. The strict definitions of mid-2000s: “Our investment mission and fiduciary duty are, in part, to maximize returns without undue risk of loss” are challenged by more comprehensive discourses: “As stewards of other peoples’ money, we are sensibly bound by a mandate to maximise risk-adjusted returns. When interpreted narrowly, such mandates preclude the involvement of institutional investors in activities that do not directly influence the portfolio’s bottom line since this could constitute a breach of fiduciary duty. However, a longer-term perspective on the task before us provides both the rationale and the imperative to act on making capitalism more inclusive”.

In contrast to asset owners, investment managers tend to define fiduciary duty more narrowly. According to BlackRock, fiduciary duty to its clients means “to protect and enhance their economic interest in the companies in which we invest on their behalf” [39]. Vanguard states that: “as a fiduciary, Vanguard is required to manage our funds in the best interests of shareholders and obligated to maximize returns in order to help shareholders meet their financial goals. It would be exceedingly difficult, if not impossible, to fulfil these obligations while managing portfolios that reflect the social concerns of all of our shareholders” [40]. Furthermore, in a 2012 statement for Pensions & Investments, Vanguard said the company does not believe that investment managers are “optimal agents to address social change.” [41]. On a system level, [34, 35] (2)) find that the index funds have strong incentives to under-invest in stewardship, and defer excessively to the preferences and positions of corporate managers. Engagements by the Big Thee only reach 10–20% of their portfolio companies. Majority of these engagements are limited to a single conversation per company per year ([34, 35] (1)).

Corporate managers are therefore increasingly accountable to distant global passive investment managers who aggregate pension assets from high-income countries by offering cost-efficient investment strategy that merely follows size (market capitalisation) of the corporations. Investment managers themselves are accountable to national and local pension funds that also aggregate retirement contributions from their jurisdictions. In summary, savers in high-income countries have surrendered the authority over their savings to distant administrators. As a result, in the case of drug development, a closely aligned network of pension administrators, investment managers and corporate executives have now acquired authority over, literally, matters of life and death.

### Including public and quasi-public institutional investors in public health ecosystem

Institutional investors have for long been conspicuously absent from the academic discourse on public health (Lexchin [45], Goldacre [49], Mirowski [46], Whitaker & Cosgrove [47]). Freudenberg within a wider discussion on industries that contribute to premature death and preventable illnesses (alcohol, firearms, pharmaceuticals, tobacco, fast food, etc.) does mention Cerberus Capital Management as an investor in gun maker Freedom Group, Inc. The private investment firm Cerberus Capital Management, he writes, is “named after the three-headed dog of Greek mythology that guards the gates of Hades” [48]. Focusing on the fund itself, however, is stopping one step short in the financial value chain. Its client base is made of: “pension plans, insurance companies, endowments, foundations, and sovereign wealth funds” [50]. Cerberus, in other words, is on a short leash. And it is instructive to point our gaze to its (often public) masters.

Economics literature, in contrast, has long recognised the importance of the market for corporate control. Management scholar Peter Drucker observed back in 1976 that “the pension funds have become America’s new ‘tycoons’ – surely the most unlikely masters any society ever had. They have attained this position without any struggle, any crisis, any major ‘problems’” [52]. Importantly, not only are the public and quasi-public institutional owners significant actors in the global capital markets. They are also political. Their boards can and do use their authority to interpret fiduciary duty and translate it into concrete investment portfolio choices. For example, the board of the California Public Employees’ Retirement System exercised its fiduciary duty to account for public health concerns when it ordered the fund to divest from all tobacco companies in 2000, overriding the recommendation of the fund’s investment staff who warned that getting rid of tobacco stocks would lower the return on investment [48].

The board of a public asset owner - a pension scheme, a sovereign wealth fund, or a central bank - is the only type of agent whose compensation is not directly dependent on maximising financial return (even at the cost of excluding certain groups of patients with life-threatening conditions from effective treatments), and whose direct mandate is to be the guardian of the beneficiaries’ welfare. As such, public asset owners boards are important actors in public health, holding financial levers over strategic healthcare decisions, such as access to medicines, but also R&D priorities.

## Conclusion

Access to medicines, which was investigated in this study, is an important public health concern, but it is not the only one. The question of access only applies to effective medicines that have already been developed. Even more important question is what kind of medicines are being developed. As with pricing, decisions on which R&D projects to pursue are guided by projected revenues. This may mean prioritising low-innovation products and reaching revenue targets through intensive marketing of those. As a result, only 1 in 10 new medicines are superior to already available treatments in terms of a statistically significant difference in primary clinical endpoints [51], while pharmaceutical companies have been consistently allocating higher budgets to marketing than to research since 1975 [64]. Nominally, these strategies in pricing and resource allocation are being pursued in the interest of the savers-shareholders, and are underwritten with their financial capital.

Financial flows directed into drug development sector via institutional governmentally-mandated mechanisms, such as retirement savings, are significant. An average high-income country citizen may be channeling comparable volumes of capital into biomedical R&D via her pension plan contributions and via her tax contributions (with no direct control in both cases). However, the standards of transparency, accountability and representation at public institutional asset owners are substantially lower than that of governments themselves. Strengthening those elements of institutional design and thus reclaiming active responsible shareholding in the interest of the patients-savers is an under-utilised, but potentially high-impact opportunity for advancing public health.

## Additional file

Additional file 1: “Medicine reimbursement denials in OECD countries due to cost considerations” is provided in a supplementary file. [61, 62, 63] (DOCX 24 kb)

## Data Availability

The datasets used and/or analysed during the current study, which are not included in this published article and its supplementary information files, are available from the corresponding author on reasonable request.

## Footnotes

1 Here “private” is used as an opposite of “public” or state-owned. Accordingly, for the purposes of this study, private companies can be listed on stock exchanges (and therefore publicly traded) or privately-held, but could nevertheless source their funding from the capital markets, for example by issuing corporate bonds.

2 To put these figures in perspective, the total wealth of the top 500 wealthiest individuals worldwide stood at $4.7 trillion at the end of 2018.

3 In the EU, out of the European Central Bank’s (ECB) $185 billion worth of corporate bond holdings, $9.3 billion is in healthcare & life sciences sectors. This includes bonds of pharmaceutical giants Bayer, Merck (Germany), Novartis, Roche and Sanofi. The Bank of Japan does not disclose sector- and firm-level statistics for its $238 billion holdings of local corporate bonds and stocks, but the total holdings imply relatively larger ownership of the local drug development firms by the central bank than in Europe [57]. In contrast, the Swiss National Bank, discloses its shareholdings in individual companies (see Table 2 for examples). Unlike the aforementioned central banks, the United States’ Federal Reserve (Fed), is currently barred by law from holding either stocks or corporate bonds. However, former Fed’s chair Janet Yellen had openly indicated in September 2016 that an extension of Fed’s mandate to cover these asset classes would be welcome [58].

4 Promotional or development banks are state-owned financial institutions promoting economic development in their jurisdictions.

5 Alexion is 94%-owned by institutional shareholders [32]. Roche’s ownership structure is more complex: while stock-exchange-listed, Roche has been majority-owned by the pool of founder’s descendants until one of them took her 5.057% of the total voting rights out of the pool, leaving the rest of the family in control of 45.01%. Further 33.3% of shares are owned by another major Swiss drug development firm, Novartis, itself stock-exchange-listed [33]. Indirect ownership of Roche via pension schemes’ investment in Novartis has not been accounted for.

6 Even in the US, a country with no universal healthcare, there is significant governmental intervention in the healthcare provision market, with over a third of the population directly insured through federally-administered Medicare and Medicaid programmes, and the rest heavily regulated.

7 Data on the proportion of total assets, which is allocated into corporate shares is from Fichtner et al. [43].

## References

[1] Dutfield G. Intellectual property rights and the life science industries: past, present and future. Singapore: World Scientific Publishing Co Pte Ltd; 2009.

[2] Moses H, Matheson DHM, Cairns-Smith S, George BP, Palisch C, Dorsey ER (2015). The anatomy of medical research. JAMA.

[3] Clark GL, Monk AHB (2017). Institutional investors in global markets.

[4] Stiglitz JE (2017). Inequality and economic growth. Polit Q.

[5] Hentov E (2015). How do sovereign wealth funds invest? A glance at SWF asset allocation.

[6] Millson B, Thiele S, Zhang Y, Dobson-Belaire W, Skinner B (2017). Access to new medicines in public drug plans: Canada and comparable countries. Annual report 2016.

[7] Medicines Australia (2017). Compare: comparison of access and reimbursement environments. A report benchmarking Australia’s access to new medicines. Edition 3, 2017.

[8] Rawson NSB. Potential impact of delayed access to five oncology drugs in Canada: Fraser Institute; 2013. https://www.fraserinstitute.org/sites/default/files/potential-impact-of-delayed-access-to-five-oncology-drugs-in-canada_1.pdf

[9] Lim CS, Lee YG, Koh Y, Heo DS (2014). International comparison of the factors influencing reimbursement of targeted anti-cancer drugs. BMC Health Serv Res.

[10] European Medicines Agency (2018). Human medicines highlights 2018.

[11] Brain tumour foundation of Canada (2018). Ask the expert: Avastin for recurrent glioblastoma Multiforme.

[12] The Pharmaceutical Management Agency (PHARMAC). Pharmaceutical schedule: PHARMAC; 2019. https://www.pharmac.govt.nz/2019/08/01/Schedule.pdf

[13] National Institute for Health and Care Excellence (NICE). Eculizumab for treating atypical hemolytic uremic syndrome, highly specialized technologies guidance: NICE; 2015. https://www.nice.org.uk/guidance/hst1/resources/eculizumab-for-treating-atypical-haemolytic-uraemic-syndrome-pdf-1394895848389

[14] Zorginstituut Nederland (National Healthcare Institute). Eculizumab (Soliris®) for the treatment of patients with paroxysmal nocturnal Haemogloninuria (PNH): Zorginstituut Nederland National Healthcare Institute; 2016. https://english.zorginstituutnederland.nl/binaries/zinl-eng/documents/reports/2016/05/13/eculizumab-soliris/Eculizumab+%28Soliris%29+%28summary+report%29.pdf

[15] Radboudumc (2017). Unique approach to orphan drugs in the Netherlands.

[16] Panteli D, Arickx F, Cleemput I, Dedet G, Eckhardt H, Fogarty E (2016). Pharmaceutical regulation in 15 European countries. Health Syst Transit.

[17] Sullivan SD, Watkins J, Sweet B, Ramsey SD (2009). Health technology assessment in health-care decisions in the United States. Int Soc Pharmacoecon Outcomes Res (ISPOR).

[18] Trullen J, Stevenson WB (2006). Strategy and legitimacy: pharmaceutical companies' reaction to the HIV crisis. Bus Soc.

[19] Canada Pension Plan Investment Board. 2019 annual report: CPPIB; 2019. http://www.cppib.com/documents/2042/CPPIB-ANNUAL-REPORT-2019-ENG.pdf

[20] BlackRock, Inc. 2019 proxy statement: BlackRock; 2019. http://ir.blackrock.com/Cache/1001251221.PDF?O=PDF&T=&Y=&D=&FID=1001251221&iid=4048287

[21] Alexion Pharmaceuticals, Inc. Proxy statement: Alexion Pharmaceuticals; 2019, 2019. https://alexion.com/2018-annual-report/assets/pdf/2019_Alexion_Proxy.pdf

[22] Alexion Pharmaceuticals, Inc. 2019 proxy statement: Alexion Pharmaceuticals; 2018. https://alexion.com/2018-annual-report/assets/pdf/ALXN_2018AR.pdf

[23] Caisse de dépôt et placement du Québec (CDPQ) (2018). Overall portfolio.

[24] ABP. Listed investments of ABP - shares & convertible bonds: ABP; 2018. https://www.abp.nl/images/listed-investments-of-abp-march.pdf. "Note that the URL may lead to a more recent version of the file than cited in the text. The publisher only maintains the most recent version online".

[25] Canada Pension Plan Investment Board. Public equity holdings as at March 31, 2017: CPPIB; 2017. http://www.cppib.com/documents/1805/foreign_publicequityholdings_Mar2018_en.htm. "Note that the URL may lead to a more recent version of the file than cited in the text. The publisher only maintains the most recent version online"

[26] British Colombia Investment Management Corporation. Investment inventory list, public equities as at March 31, 2017 (unaudited): British Colombia Investment Management Corporation; 2017. https://www.bci.ca/wp-content/uploads/2018/02/investmentinventory2017-publicequities.pdf

[27] New Zealand Superannuation Fund. 2017 equity listing: NZ Super Fund; 2018. https://www.nzsuperfund.co.nz/publications/annual-equity-listings

[28] Strathclyde Pension Fund. Strathclyde pension fund assets: Strathclyde Pension Fund; 2018. http://www.spfo.org.uk/CHttpHandler.ashx?id=30963&p=0

[29] West Yorkshire Pension Fund. WYPF valuation 2017: WYPF; 2017. http://www.wypf.org.uk/Member/Investments/InvestmentPortfolio/2017/WYPFValuation2017.aspx

[30] US Securities and exchange commission (SEC) (2017). Swiss National Bank, filing details.

[31] European Central Bank (ECB). Asset purchase Pogrammes: European Central Bank; 2017. https://www.ecb.europa.eu/mopo/implement/omt/html/index.en.html#cspp

[32] NASDAQ (2018). Alexion Pharmaceuticals, Inc. institutional ownership.

[33] Roche. Annual report 2017: Roche; 2018. https://www.roche.com/dam/jcr:78519d71-10af-4e02-b490-7b4648a5edb8/en/ar17e.pdf

[34] Bebchuk L, Hirst S. The specter of the Giant three, vol. 99. Boston: Boston University Law Review; 2019.

[35] Bebchuk L, Hirst S. Index funds and the future of corporate governance: theory, evidence, and policy, vol. 119. New York: Columbia Law Review. 2019b. https://papers.ssrn.com/sol3/papers.cfm?abstract_id=3282794

[36] UK Law Commission. Fiduciary duties of investment intermediaries: Web ISBN 9781474107648, UK Law Commission; 2015. http://www.lawcom.gov.uk/app/uploads/2015/03/lc350_fiduciary_duties.pdf

[37] Sullivan R, Martindale W, Feller E, Bordon A, Boaretto A. Fiduciary duty in the 21ST century: UNEP finance initiative, the PRI, the UN global compact and the UNEP inquiry; 2015. http://www.unepfi.org/fileadmin/documents/fiduciary_duty_21st_century.pdf

[38] Hart O, Zingales L (2017). Companies should maximize shareholder welfare not market value. J Law Finance Account.

[39] BlackRock Inc. Global corporate governance and engagement principles: BlackRock Inc; 2014. https://www.sec.gov/Archives/edgar/data/1053988/000119312515334862/d92408dex99corpgov.htm

[40] Vanguard Investments (2018). Vanguard's view: social concerns and investing.

[41] Olsen K (2012). Public pension plans Mull purging gun Investments.

[42] California State Teachers’ Retirement System. Comprehensive annual financial report for the fiscal year ended June 30, 2017: California State Teachers’ Retirement System; 2017. https://www.calstrs.com/sites/main/files/file-attachments/cafr2017.pdf

[43] Fichtner J, Heemskerk E, Garcia-Bernardo J (2017). Hidden power of the big three? Passive index funds, re-concentration of corporate ownership, and new financial risk. Bus Polit.

[44] Organisation for Economic Co-operation and Development (OECD). Pension Markets in Focus 2017: OECD; 2017. http://www.oecd.org/pensions/private-pensions/Pension-Markets-in-Focus-2017.pdf

[45] Lexchin J (2016). Private profits versus public policy: the pharmaceutical industry and the Canadian state.

[46] Mirowski P. Science-Mart: privatizing American science. Cambridge, Massachusetts: Harvard University Press; 2011.

[47] Whitaker R, Cosgrove L (2015). Psychiatry under the influence.

[48] Freudenberg N (2014). Lethal but legal: corporations, consumption, and protecting public health.

[49] Goldacre B (2012). Bad pharma: how drug companies mislead doctors and harm patients (fourth estate).

[50] Cerberus Capital Management. ESG report 2017: Cerberus Capital Management; 2017. Available from: https://www2.deloitte.com/content/dam/Deloitte/ky/Documents/about-deloitte/aiml-liquidation/Cerberus_ESG-Report-January2018.pdf

[51] Van-Luijn JCF, Gribnau FWJ, Leufkens HGM (2010). Superior efficacy of new medicines?. Eur J Clin Pharmacol.

[52] Drucker PF. The pension fund revolution. New Brunswick: Transaction Publishers; 1992.

[53] World Bank (2017). Market capitalization of listed domestic companies (current US$).

[54] Rosenthal SM, Austin LS. The dwindling taxable share of U.S. corporate stock: special report: Tax Policy Center; 2016. http://www.taxpolicycenter.org/publications/dwindling-taxable-share-us-corporate-stock/full

[55] International Monetary Fund (IMF). Global financial stability report: grappling with crisis legacies: IMF; 2011. https://www.imf.org/~/media/Websites/IMF/imported-full-text-pdf/external/pubs/ft/gfsr/2011/02/pdf/_text.ashx

[56] Peetz D, Murray G. The Financialization of global corporate ownership. In: Murray G, Scott J, editors. Financial elites and transnational business: who rules the world? Cheltenham: Edward Elgar Publishing; 2012.

[57] Bank of Japan. Bank of Japan Accounts: Bank of Japan; 2018. https://www.boj.or.jp/en/statistics/boj/other/acmai/release/2018/ac180410.htm/

[58] Lange J, Dunsmuir L. Yellen says fed purchases of stocks, corporate bonds could help in a downturn: Reuters, Business News; 2017. https://www.reuters.com/article/us-usa-fed-yellen-purchases/yellen-says-fed-purchases-of-stocks-corporate-bonds-could-help-in-a-downturn-idUSKCN11Z2WI

[59] DiMasi JA, Grabowski HG, Hansen RW (2016). Innovation in the pharmaceutical industry: new estimates of R&D costs. J Health Econ.

[60] Gagnon MA, Hebert G. The economic case for universal Pharmacare costs and benefits of publicly funded drug coverage for all Canadians: ISBN 978–1–926888-13-2, Canadian Centre for Policy Alternatives (CCPA) and Institut de recherche et d’informations socio-economiques (IRIS); 2010. https://nursesunions.ca/wp-content/uploads/2017/07/universal-pharmacare-report-e.pdf

[61] Pan Canadian Oncology Drug Review (PCODR). PCODR expert review committee PERC final recommendation: PCODR; 2015a. https://cadth.ca/sites/default/files/pcodr/pcodr-avastincc-fn-rec.pdf

[62] Pan Canadian Oncology Drug Review (PCODR). PCODR expert review committee PERC final recommendation: PCODR; 2015b. https://cadth.ca/sites/default/files/pcodr/pcodr_bevacizumab_avastin_oc-fn_rec.pdf

[63] Pan Canadian Oncology Drug Review (PCODR). PCODR expert review committee PERC final recommendation: PCODR; 2016. https://cadth.ca/sites/default/files/pcodr/pcodr_bevacizumab_avastin_proc_fn_rec.pdf

[64] Weiss D, Naik P, Weiss R (2009). The 'big pharma' dilemma: develop new drugs or promote existing ones?. Nat Rev Drug Discov.

