# Supplementary material for: Pension and state funds dominating biomedical R&D investment: fiduciary duty and public health
Source: Global Health. 2019 Nov 6;15:55. doi: 10.1186/s12992-019-0490-x (PMC6833262; doi:10.1186/s12992-019-0490-x)
Supplement: Supplementary file 1 — “Medicine reimbursement denials in OECD countries due to cost considerations” is provided in a supplementary file. [61, 62, 63] (DOCX 24 kb) [file 12992_2019_490_MOESM1_ESM.docx]

Additional file 1

Appendix for

Pension and state funds dominating biomedical R&D investment: Fiduciary duty and public health

**Medicine reimbursement denials in OECD countries due to cost considerations**

Formal appraisal procedures for new medicines have been instituted for cost controlling purposes and can result in restrictive treatment guidelines and limitations on the use of expensive drugs. Some jurisdictions employ a fixed and explicit threshold for cost per QALY, such as €80 000 in the Netherlands, three times GDP per capita in Poland, and €18 000 in Slovakia. Others such as Norway and Sweden, use variables or no explicit thresholds where the willingness to pay depends on the severity of the disease area and level of unmet need (WHO, 2015). Therefore, a medicine’s marketing authorisation does not guarantee patient access, even in OECD countries. Regulatory decisions across OECD on reimbursement of medicines investigated in this study are provided below.

BEVACIZUMAB - Canada: denial of coverage

Final Recommendation of 23 March 2015:

“pERC [Pan Canadian Oncology Drug Review (PCODR) Expert Review Committee] concluded that bevacizumab plus chemotherapy for the treatment of patients with metastatic, persistent, or recurrent carcinoma of the cervix was not cost-effective at the submitted confidential price.” (CADTH, 2015a).

Final Recommendation of 4 June 2015:

“Upon reconsideration of the pERC Initial Recommendation, pERC considered feedback received from the patient advocacy group that patients value treatment with bevacizumab plus carboplatin and paclitaxel and that funding should be provided regardless of its cost-effectiveness. The Committee noted that it is required to make conclusions around cost-effectiveness as part of its Deliberative Framework, and importantly, this allows the provinces to make informed decisions regarding funding relative to other cancer therapies. Therefore, pERC concluded that the original conclusion was still appropriate and, specifically that bevacizumab plus carboplatin and paclitaxel may not be cost-effective at the submitted price [for the front-line treatment of epithelial ovarian, fallopian tube or primary peritoneal cancer patients with high risk of relapse (stage III sub-optimally debulked, or stage III unresectable, or stage IV patients]” (CADTH, 2015b).

Final Recommendation of 5 May 2016:

“[pERC] concluded that, at the submitted price for bevacizumab, bevacizumab plus chemotherapy is not cost-effective relative to chemotherapy alone in patients with platinum-resistant, recurrent epithelial ovarian, fallopian tube or primary peritoneal cancer who have received no more than 2 prior anticancer regimens.” (CADTH, 2016a).

BEVACIZUMAB - United Kingdom: denial of coverage

“Not recommended” for colorectal cancer (NICE, 2007, 2010 & 2012); advanced and/or metastatic renal cell carcinoma (NICE, 2009); metastatic breast cancer (NICE, 2011); advanced ovarian cancer (NICE 2013a & 2013b).

BEVACIZUMAB - New Zealand: denial of coverage

Included in Pharmaceutical Schedule (Section H) of medicines reimbursed by the Pharmaceutical Management Agency only for use in ophthalmology, not as a cancer treatment (PHARMAC, 2018b).

BEVACIZUMAB - France: covered

HAS “recommends inclusion on the list of reimbursable products for hospital use” for the first-line management of advanced and/or metastatic renal cell carcinoma, metastatic colorectal cancer, advanced stage ovarian cancer and persistent, recurrent or metastatic cervical cancer (HAS, 2016).

ECULIZUMAB - Netherlands: denial of coverage

“Based on necessity, effectiveness, cost-effectiveness and feasibility (the package criteria), eculizumab should no longer be reimbursed from the basic health insurance, unless there is clear insight into its cost-effectiveness (and in a case of unfavourable cost-effectiveness) transparency regarding how the price was determined.” (Zorginstituut Nederland, 2016)

ECULIZUMAB - France: covered

ASFOTASE ALFA - Canada: limited coverage

*Alexion Pharmaceuticals’ another breakthrough medicine, asfotase alfa, indicated for a rare, potentially fatal condition hypophosphatasia (HPP). It was discovered at the University of Montreal, then initially developed by Montreal-based Enobia Pharma, which was subsequently purchased by Alexion in 2011* (Grant, 2017). *Asfotase alfa is a lifetime therapy; it is not a cure, but can improve the quality of life.*

CADTH recommended reimbursement only if a “Patient is not an adult at the time treatment is initiated” (CADTH, 2016b), thereby denying access to adults.

ASFOTASE ALFA - United Kingdom: full coverage

While initially NICE “felt the benefits of the drug were too uncertain in the whole population in relation to its very high price for them to be able to recommend it for any but the most seriously affected”, from July 2017 recommendation for reimbursement was extended to both children and adults (NICE, 2017).
